# Targeting Caspase-3 as Dual Therapeutic Benefits by RNAi Facilitating Brain-Targeted Nanoparticles in a Rat Model of Parkinson’s Disease

**DOI:** 10.1371/journal.pone.0062905

**Published:** 2013-05-13

**Authors:** Yang Liu, Yubo Guo, Sai An, Yuyang Kuang, Xi He, Haojun Ma, Jianfeng Li, Jing Lv, Ning Zhang, Chen Jiang

**Affiliations:** 1 Key Laboratory of Smart Drug Delivery, Ministry of Education and PLA, School of Pharmacy, Fudan University, Shanghai, People’s Republic of China; 2 Department of Life Sciences and Technology, Caliper-a PerkinElmer Company, Alameda, California, United States of America; University of Florida, United States of America

## Abstract

The activation of caspase-3 is an important hallmark in Parkinson’s disease. It could induce neuron death by apoptosis and microglia activation by inflammation. As a result, inhibition the activation of caspase-3 would exert synergistic dual effect in brain in order to prevent the progress of Parkinson’s disease. Silencing caspase-3 genes by RNA interference could inhibit the activation of caspase-3. We developed a brain-targeted gene delivery system based on non-viral gene vector, dendrigraft poly-L-lysines. A rabies virus glycoprotein peptide with 29 amino-acid linked to dendrigraft poly-L-lysines could render gene vectors the ability to get across the blood brain barrier by specific receptor mediated transcytosis. The resultant brain-targeted vector was complexed with caspase-3 short hairpin RNA coding plasmid DNA, yielding nanoparticles. *In vivo* imaging analysis indicated the targeted nanoparticles could accumulate in brain more efficiently than non-targeted ones. A multiple dosing regimen by weekly intravenous administration of the nanoparticles could reduce activated casapse-3 levels, significantly improve locomotor activity and rescue dopaminergic neuronal loss and in Parkinson’s disease rats’ brain. These results indicated the rabies virus glycoprotein peptide modified brain-targeted nanoparticles were promising gene delivery system for RNA interference to achieve anti-apoptotic and anti-inflammation synergistic therapeutic effects by down-regulation the expression and activation of caspase-3.

## Introduction

Parkinson’s disease (PD) is classically characterized as loss of striatal dopaminergic neurons [1]. However, PD has a complex pathophysiology that hasn’t been fully understood and involves multiple brain structures and signaling pathways [2]. Previous approaches usually focus on the selection of a single therapeutic target. The most straightforward way is to increase dopamine levels by dopamine replacement therapy or introduce key enzymes involved in dopamine metabolism [3], [4]. Neurotropic factors are also used to prevent the death of dopaminergic neurons [5]. Epidemiological studies suggest that environmental toxins exposure is closely associated with an increased risk of developing PD [6]. Neurotoxins such as rotenone could induce neurotoxicity via the activation of caspase-3 in both in vitro and in vivo experiments [7], [8].

Human postmortem studies have suggested that nigral dopamine (DA) neurons die by apoptosis in PD [9]. Unfortunately, the obvious symptoms of PD do not develop until there is an estimated 70–80% reduction in the DA content of the caudate putamen and an estimated 50–60% loss of DA neurons in the substantia nigra [10]. Based on the above evidence, preventing neuron death at the early stage of the disease would be one of the promising strategies that could treat or delay the progress of the disease. Studies suggest caspase-3 plays a central role in the process of neuron apoptosis [11]. Thus, caspase-3 may constitute an attractive target for anti-apoptotic therapy in PD.

Neuron loss is usually accompanied with the death of astrocytes and activation of the microglia. Recent researches reveal that activation of microglia and inflammation-mediated neurotoxicity are crucial in the pathogenesis of PD [12]. Uncontrolled and over-activated microglia can trigger neurotoxicity and induce neuron death. Inhibition of the caspase-3 pathway could effectively block microglia activation and prevent neuron death in order to play neuro-protective effect. Thus, caspase-3 may also constitute an attractive target for anti-inflammation therapy in PD. Taken together, inhibition the activation of caspase-3 would exert synergistic anti-apoptotic and anti-inflammation dual effect in neurons and microglia, respectively, in brain contributing to prevent the progress of PD.

RNA interference (RNAi) could induce specific post-transcriptional gene silencing. It provides a promising approach to down-regulate of caspase-3 expression for the gene therapy in PD. However, many natural barriers especially the blood-brain barrier (BBB) have to be overcome for safe and efficient delivery of therapeutic siRNA or short hairpin RNA (shRNA) to brain [13]. Meanwhile, the stability of siRNA and shRNA during the process of delivery should be also taken special consideration and avoid degradation by enzymes in blood or extracellular environments. The shRNA encoding plasmid is thought to be more stable than siRNA or shRNA for *in vivo* applications and employed in numerous studies [14]. Thus, in order to achieve successful gene therapy for PD, it is crucial to solve the following problem: how to deliver caspase-3 shRNA encoding plasmid across the BBB to brain parenchyma cells (neurons and microglia) efficiently and specifically?

Dendrigraft poly-L-lysines (DGLs) have emerged as a new kind of synthetic polymers consisted of lysine. They have been employed as non-viral gene vector in this study due to their degradability and rich external amino groups which could encapsulate the negatively charged plasmid DNA [15], [16]. PEGylation on surface of DGLs would further extend the half-life of the gene delivery system, lower surface charge and reduce non-specific uptake by non-targeted tissues. The active brain-targeted modification to DGLs could render them the ability to get across the BBB by specific receptor mediated transcytosis and decrease the accumulation in other peripheral organs. The rabies virus glycoprotein peptide (RVG29), a 29 amino-acid peptide derived from the rabies virus glycoprotein, is found to bind the nicotinic acetylcholine receptor (nAchR) that is widely expressed in the brain including brain capillary endothelial cells, the main components of the BBB [17].

The high biocompatibility of DGLs allows multiple doing administration to ensure the sustained down-regulation of the caspase-3 and maintain it at a low level since the progress of PD is chronic and long-term. As a result, a multiple dosing regimen by weekly intravenous administration is designed in this study to achieve prompt intervention of the PD pathology at the early stage. The brain-targeted peptide RVG29 was modified to DGLs as non-viral gene vector through bi-functional PEG linker. The resultant DGLs-PEG-RVG29 (DPR) was further complexed with plasmid DNA encoding caspase-3 shRNA (pshC-3), the gene drugs in this study, yielding DPR/pshC-3 nanoparticles (NPs). The characteristics of the NPs were investigated. The accumulation efficiency in brain of targeted NPs was compared to non-targeted ones by *in vivo* imaging analysis. The pharmacodynamics of the NPs applied in rotenone-induced PD rat model was evaluated by behavioral tests, detection of caspase-3 level and tyrosine hydroxylase (TH) positive neuron immunostaining. Furthermore, the anti-apoptotic and anti-inflammation dual therapeutic mechanisms targeted to down-regulation of caspase-3 by the NPs were further investigated.

## Materials and Methods

### ShRNA Encoding Plasmid Construction and Preparation

The caspase-3 shRNA encoding plasmid (pshC-3) and scramble shRNA encoding plasmid (pshSc) were constructed into pGPU6/Neo plasmid vector by Genepharma Ltd. Co. Initially, three different caspase-3 shRNA sequence were designed according to the results by applying specific software. The percentage of gene silencing efficiency of all the three shRNA encoding plasmids as well as scramble shRNA encoding plasmid was evaluated (specifically described in Supporting Information S1 and Figure S2 in Supporting Information S1). The most efficient sense strand of caspase-3 shRNA sequence was 5′- caccGCCGAAACTCTTCATCATTCATtcaagagATGAATGATGAAGAGTTTCGGCttttttg-3′. Uppercase letters indicated 20-nucleotide (nt) caspase-3 target sequences and lowercase letters indicated hairpin and sequences necessary for the directional cloning into the plasmid vector. Scramble shRNA encoding plasmid used as negative control had random sequences (sense strand 5′-caccGTTCTCCGAACGTGTCACGTcaagagattacgtgACACGTTCGGAGAAttttttg-3′) with no homology. The plasmid DNA (pDNA) grown in *E*. *coli* was isolated with the Endo-Free Plasmid Mega Kit (Qiagen GmbH, Germany). The purity was confirmed by spectrophotometry (A260/A280), and DNA concentration was measured by UV absorption at 260 nm.

### *In vivo* Distribution of the NPs

Male Sprague-Dawley (SD) rats weighing 250–300 g of two months old were purchased from the Sino-British SiPPR/BK Lab. Animal Ltd. and maintained under standard housing conditions. Balb/c mice weighing 20 g of two weeks old were provided by Caliper Lifescience (PerkinElmer Company). This study was carried out in strict accordance with the recommendations by the ethics committee of animal welfare in Fudan University. The protocol was approved by the ethics committee of animal welfare in Fudan University. Most operations were performed under chloral hydrate or isoflurane anesthesia, and all efforts were made to minimize suffering.

For in vivo imaging, the BODIPY (ex/em = 650/665 nm) labeled DPR/DNA (reaction ratio: BODIPY/DGLs = 4/1, mol/mol) NPs (100 µg/20 g mouse, calculated by DGLs) were injected intravenously through the tail vein into a Balb/c mouse while the DP/DNA (DGLs-PEG/DNA) NPs was injected as control. The mice under anesthesia were imaged with the 650 nm excitation and 665 nm emission filters using the IVIS Spectrum CT imaging system at different time points after injection.

For the analysis of the NPs distribution in brains, rats were administrated with BODIPY labeled DPR/DNA NPs (600 µg/250 g rat, calculated by DGLs) as well as DP/DNA NPs. Rats with different NPs administration at 2 h were anaesthetized by 10% chloral hydrate on specified days and perfused transcardially with saline followed by 4% paraformaldehyde/PBS pH 7.4. The brains were rapidly removed and post-fixed for 24 h, then transferred to PBS containing 30% sucrose at 4°C until subsidence. Coronal brain sections were made at a thickness of 30 µm and processed for brain capillary endothelial cells marker (Factor VIII) and neuron marker (neurofilament) immunofluorescence staining using anti-Factor VIII antibody (1∶1000; Abcam, USA) and anti-Neurofilament H (RMdO 20) antibody (1∶200; CST, USA). Alexa Fluor 555 conjugated secondary antibodies (1∶1000; CST, USA) were also used. Nucleus was stained with 300 nM DAPI (4,6-diamidino-2-phenylindole; Molecular Probes, USA) for 10 min at room temperature. The sections were observed under fluorescence microscope.

### Transport Studies of NPs Across BCECs Monolayer

Brain capillary endothelial cells (BCECs) were kindly provided by Prof. J. N. Lou (the Clinical Medicine Research Institute of the Chinese-Japanese Friendship Hospital) [18]. The specific culture condition was described in **supporting information**. BCECs were seeded at the density of 7*10^4^ cells/cm^2^ onto polycarbonate 24-well Transwell filters (mean pore size: 1.0 µm; surface area: 0.33 cm^2^; FALCON Cell Culture Insert, Becton Dickinson Labware, USA). The cell monolayer integrity was monitored using an epithelial voltohmmeter to measure the transendothelial electrical resistance (TEER) after 2–3 days’ culture. Only cell monolayers with TEER exceeding 200 Ωcm^2^ were selected for this experiment. DGLs was radio-labeled with radioactivity of 1 mCi ^125^I/mg DGLs. 800 µl fetal bovine serum (FBS)-free medium was added to the acceptor chamber at time 0. At the same time, non-targeted and brain-targeted NPs were added to donor chamber in 300 µl fetal bovine serum (FBS)-free medium, respectively, with a final amount of 20 µg DGLs/well. The incubation was performed at 37°C on a rocking platform at 50 rpm. The radioactivity of ^125^I in each aliquot was assessed using a γ-counter. The apparent permeability (Papp) was calculated as follows:
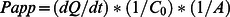
where dQ/dt is the permeability rate (nmol/s), C_0_ is the initial concentration (nmol/ml) in the donor chamber, and A is the surface area (cm^2^) of the membrane filter. The TEER of BCECs monolayers were measured after the NPs treatment to monitor the integrity of monolayers.

### Rotenone Treated Rats and NPs Administration Regimen

Rotenone (Sigma-Aldrich, USA) emulsified in corn oil at 1.25 mg/ml was given intraperitoneally to SD rats once a day at 2.5 mg/kg for various lengths of time ranging from 10 to 45 days. Animals were weighed every three days to indicate basic status.

Rotenone treated rats were administrated intravenously with DPR/pshSc or DPR/pshC-3 NPs (600 µg/rat, calculated by DGLs) weekly on the 7th, 14th, 21st and 28th day. Meanwhile, the saline was injected as negative control. The rats were sacrificed on the 10th, 20th, 25th and 45th day, respectively. The regimen was shown in Table 1. The level of activated caspase-3 and related immunostaining were evaluated in one injection groups (on the 10th day), two injections groups (on the 20th day), three injections groups (on the 25th day) and four injections groups (on the 45th day).

**Table 1:** The regimen of multiple dosing administration in rotenone-treated rats.

Group name Days	7	10	14	20	21	25	28	45
Oil								x
+Saline	iv	x	iv	x	iv	x	iv	x
+DP/pshC-3	iv		iv		iv		iv	x
+DPR/pshSc	iv	x	iv	x	iv	x	iv	x
+DPR/pshC-3	iv	x	iv	x	Iv	x	iv	x

iv: intravenous administration of the NPs/saline.

x: sacrifice for mRNA, western blot, immunofluorescence and immunohistochemistry assays.

+: rotenone treated rats with corresponding iv administration;

Oil: oil treated rats without saline or NPs administration.

### Real-time Quantitative RT-PCR Analysis of Caspase-3 mRNA

For quantitative assessment of relative mRNA levels, total RNA from substantia nigra area in different groups was prepared using Trizol LS reagent (Invitrogen, USA) following manufacturer instructions. The RT-PCR analysis were done by Shanghai GenePharma Co. Ltd. Values obtained were normalized to the amount of GAPDH. The primer and reverse primer: Caspase-3∶5′ TGTATGCTTACTCTACCGCACCCG, 5′ GCGCAAAGTGACTGGATGAACC; GAPDH: 5′ GATGACATCAAGAAGGTGGTGA, 5′ ACCCTGTTGCTGTAGCCATATTC. Briefly, 5 µl of template RNA were denatured (95°C/3 min). Reactions were performed using 50 nM primer. Amplifications were done in a Stratagene MX3000P Real-time PCR machine (Stratagene, US) with the following cycle parameters: 45 min at 42°C (reverse transcription), 10 min at 85°C (RT inactivation and PCR polymerase activation), 40 cycles of 95°C/12 sec, and 62°C/60 sec.

### Determination of Activated Caspase-3 by Western Blot

Rats with different rotenone treatment duration and NPs administration were sacrificed. Brain tissues were lysed in RIPA lysis buffer containing protease inhibitor PMSF. The samples containing 50 µg proteins were separated by SDS-PAGE and transferred onto PVDF membranes at 150 mA for 60 min (Mini-PROTEAN, Bio-Rad). Primary antibodies used for western blot analysis were anti-cleaved caspase-3 (Asp175) (1∶1000; CST, USA) and anti-GAPDH antibody (1∶200; Beyotime, China). Then, the PVDF membranes were incubated with horseradish peroxidase–conjugated secondary antibodies. The results were detected by chemiluminescence using SuperSignal West Pico kit (Pierce Biotech, USA).

### Determination of Activated Caspase-3 by Immunofluorescence

Rats with different rotenone treatment duration and NPs administration were anaesthetized by 10% chloral hydrate on specified days and perfused transcardially with saline followed by PBS pH 7.4 and 4% paraformaldehyde. The brains were rapidly removed and post-fixed for 24 h, then transferred to PBS containing 30% sucrose at 4°C until subsidence. Coronal brain sections were made at a thickness of 30 µm and processed for activated caspsase-3 immunofluorescence staining using anti-cleaved caspase-3 (Asp175) antibody (1∶200; CST, USA). Alexa Fluor 555 conjugated goat anti-rabbit secondary antibody (1∶1000; CST, USA) was used for fluorescence microscope (DMI 4000B, Leica, Germany) observation. For quantitative analysis of the percentage of activated caspase-3 positive signals, coronal section images were counted using software Image-Pro Plus Version 6.0.

### TH Positive Immunohistochemistry

Float serial coronal sections were incubated in 0.25% Triton X-100 for 30 min followed by 0.3% hydrogen peroxide for 15 min, then blocked with 5% BSA for 2 h. After that, the sections were incubated with anti-TH monoclonal antibody (1∶200; Millipore, USA) overnight at 4°C. Subsequently, the sections were incubated with secondary biotinylated antibody for 1 h at room temperature, followed by incubation with a streptavidinbiotinylated-horseradish peroxidase complex, following the instructions of the ABC kit. The staining was developed with DAB as the chromogen. Representative images were taken using a microscope (DMI 4000B, Leica, Germany).

### Stereological Counting

Unbiased counting of TH-immunoreactive dopaminergic neurons within the substantia nigra (SN) was performed as described previously [19], [20]. For each rat brain, four selected representative sections of the SN were analyzed. The numbers of TH immunoreactive cells in the SN pars compacta (SNpc) were counted using an optical fractionator method. This stereological method of cell counting was not affected by either the reference volume (SNpc) or the size of the counted elements. The analysis was done by an individual completely blind to the treatment group of each brain.

### Behavioral Analysis (open-field test)

Locomotor activity was measured on 15th, 25th, 35th and 45th day from the 1st day of rotenone treatment. Each rat was placed in the center of an open field (80 cm×48 cm bottom, 50 cm wall around). The floor of the field was divided into 15 small equal-sized squares (16 cm×16 cm). The movement and behavior of the rats were observed for 5 min [21]. Two main parameters were measured in this experiment: line crossing (number of lines crossed) and inactive sitting (time in seconds). Evaluation was done under double-blind condition.

### Apoptosis Analysis by TUNEL

Terminal deoxynucleotidyltransferase-mediated dUTP nick end-labeling staining (TUNEL) was used to evaluated the degrees of apoptosis in brains. Coronal brain sections from different groups were proceeded for TUNEL staining using in situ cell apoptosis detection kit (Keygen, China) according to the manufacturer’s instructions.

### Determination of TNF-α Level by ELISA

Rats with different rotenone treatment duration and NPs administration were sacrificed. Brain tissues were lysed in RIPA lysis buffer containing protease inhibitor PMSF. The supernatant TNF-α level was quantified using ELISA according to the manufacturer’s instructions (R&D Systems, USA). The results were measured at 570 nm using a microplate reader (BIO-TEK, USA).

### NO Assay

Brain tissues were snap frozen in liquid nitrogen and stored at −80°C until further use. Production of nitric oxide (NO) was determined by measuring the accumulated level of nitrite (an indicator of NO) in the brain supernatant after different days of rotenone treatment using a colorimetric reaction with Griess reagent. Briefly, 100 µL of supernatant were mixed with 100 µL Griess reagent [0.1% N-(1-naphthyl) ethylenediaminedihydrochloride, 1% sulfanilamide, and 2.5% H_3_PO_4_] [22]. After incubation at room temperature in the dark for 10 min, total nitrites were measured spectrophotometrically at 540 nm using a microplate reader (Synergy 2, Biotek, USA). The concentration of nitrite in the sample was determined from a NaNO_2_ standard curve.

### Statistical Analysis

The data are presented as mean±SD. The statistical significance was determined using student’s t-test and analysis of variance (ANOVA).

## Results

### *In vivo* Distribution of the NPs

The successful preparation and characterization of the brain-targeted vector and nanoparticles wereshown in Fig. S1A–D, Fig. S3 and information in Supporting Information S1.

**Figure 1:**
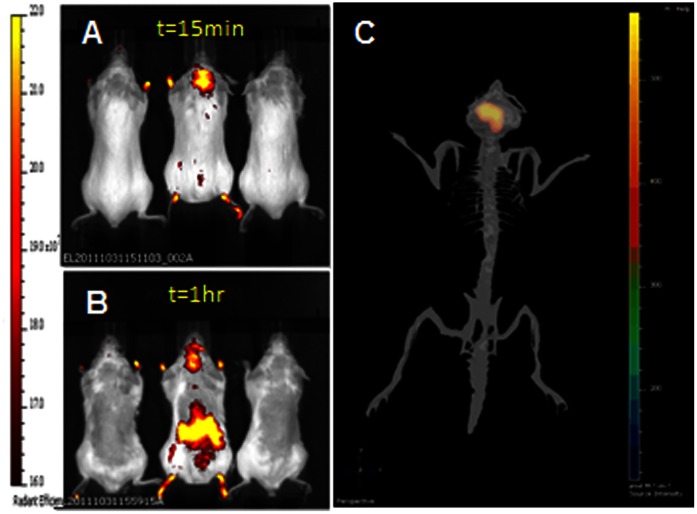
*In vivo* imaging of fluorescent labeled DPR/DNA NPs. (**A**) *In vivo* distribution in mice with different treatment (Left: DGLs/DNA NPs; Middle: DPR/DNA NPs; Right: without treatment) at 15 min after intravenous administration in Balb/c mice. (**B**) *In vivo* distribution in mice with different treatment (Left: DGLs/DNA NPs; Middle: DPR/DNA NPs; Right: without treatment) at 1 h after intravenous administration in Balb/c mice. (**C**) 3D fluorescent localization in DPR/DNA NPs injected mouse skeleton with CT scanning.

**Fig. 1A and B** showed the BODIPY-labeled fluorescent signal was obviously accumulated in mice brain administrated with the DPR/DNA NPs intravenously. Meanwhile, the fluorescence in the brain of the DGLs/DNA NPs injected mice was too weak to be detected which was similar to that in untreated control mouse. The DPR/DNA NPs showed a rapid and specific accumulation in brain at 15 min after administration. While, the accumulation in liver increased at 1 h after administration. After processing the 3D analysis in brain area of DPR/DNA NPs injected mice, the signal was found to co-locate well in the brain (**Fig. 1C**). In our previous study [23], the principal organs including brain, heart, liver, spleen, lung and kidney were removed for ex vivo imaging analysis. The signals were mainly accumulated in liver, spleen and kidney. The signals reached the peak at 1 h after injection.

### Tracing the DPR/DNA NPs in the Brain

**Figure 2:**
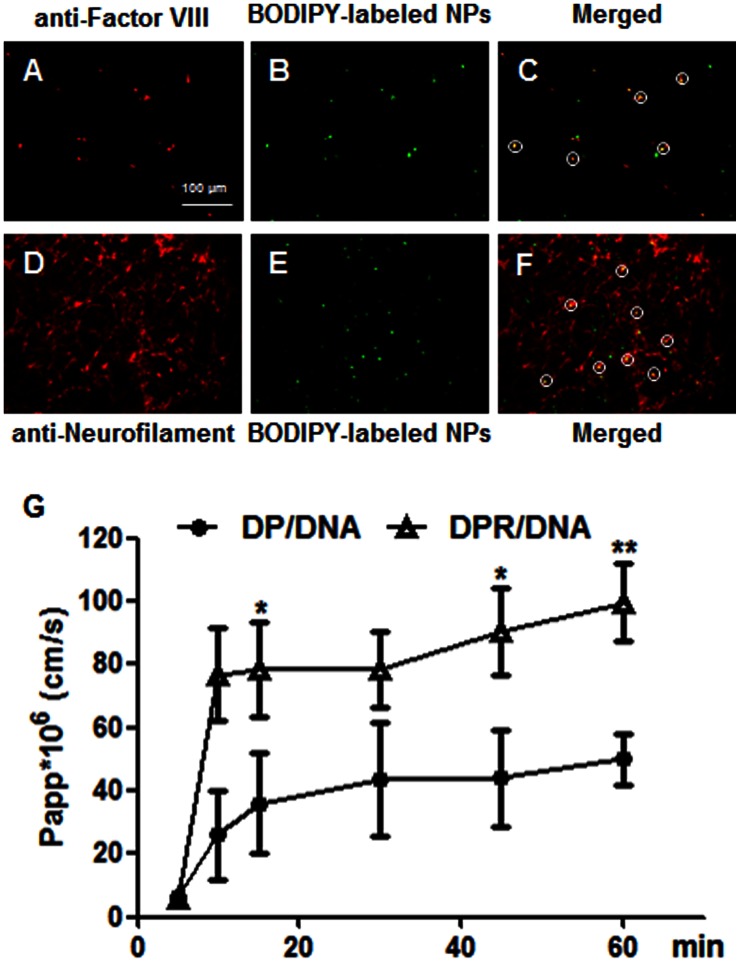
The localization of NPs in midbrain and the permeability of ^125^I-labeled NPs across BCECs monolayer. Brain sections of NP injected rat were immunostained with (**A**) anti-Factor VIII polyclonal antibody labeled brain capillaries and (**D**) anti-Neurofilament monoclonal antibody labeled neurons. (**B**) and (**E**) were images of NPs distribution. (**C**) was the merged image of (**A**) and (**B**), while (**F**) was that of (**D**) and (**E**). The circles indicated the colocation of NPs and related labeled cells. Red: Alexa Fluor 555 labeled secondary antibody; Green: BODIPY labeled DPR/DNA NPs; Yellow: Merged signal of red and green. Original magnification: ×200. (**G**) The permeability of ^125^I-labeled NPs across BCECs monolayer. Significance: *, p<0.05; **, p<0.01, significance represents DPR/DNA NPs vs. DP/DNA NPs.

**Figure 3:**
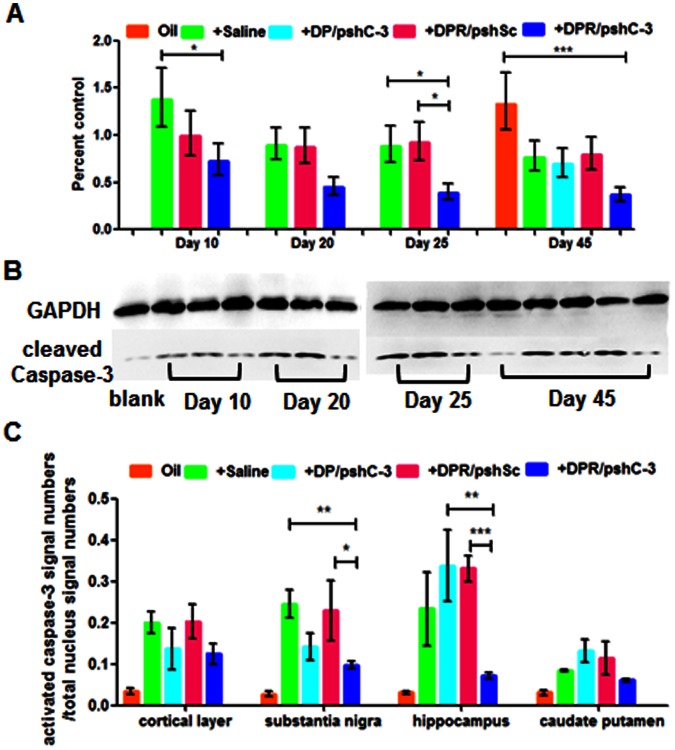
Evaluation of activated caspase-3 in rotenone induced Parkinson’s disease rat model. (**A**) Caspase-3 mRNA silencing percentage by RT-PCR in brains at various days of rotenone treatment in rats with different NPs. Data are expressed as mean±S.E.M (n = 3). Significance: *p<0.05; ***p<0.001, significantly different as compared between two groups. (**B**) Activated caspase-3 comparison by western blot in brains at various days of rotenone treatment in rats with different NPs. (**C**) Quantitative evaluation of activated caspase-3 positive immunofluorescence at cortical layer, substantia nigra, hippocampus and caudate putamen and in rats with rotenone treatment for 45 days.

The distribution of BODIPY-labeled nanoparticles in brain sections of rats 2 hours after injection was observed with anti-Von Willebrand Factor polyclonal antibody to stain the brain capillaries and anti-Neurofilament monoclonal antibody to stain the neurons. As shown in **Fig. 2A–F**, the fluorescent signals (green) of BODIPY-labeled nanoparticles was partially co-located with Alexa Fluor 555 labeled anti-Von Willebrand Factor antibody (**Fig. 2A–C**) and Alexa Fluor 555 labeled anti-Neurofilament antibody (**Fig. 3D–F**), respectively. This result indicated that the RVG29 modified brain-targeted NPs could cross the brain capillaries and enter the brain. Some of the NPs could be uptake by neurons. And some of NPs would be uptake by other brain parenchyma cells including microglia.

### Transport Studies of NPs across BCECs Monolayer

The results of transport studies of DP/DNA and DPR/DNA NPs were shown in **Fig. 2G**. Papp of DPR/DNA NPs was significantly higher than that of DP/DNA NPs after 10 min. It indicated that DPR/DNA NPs could get across the BBB efficiently with the modification of RVG29. The transendothelial electrical resistance (TEER) showed no significant difference from that of controls (data not shown) verifying the integrity of BCECs monolayer during the experiments.

### Evaluation of Caspase-3 Activation in Rotenone Induced Parkinson’s Disease Rat Model

The regimen of multiple dosing administration of gene loading NPs in rotenone-treated rats was shown in **Table 1**. The NPs were injected intravenously every week on the 7^th^, 14^th^, 21^th^ and 28^th^ day of rotenone treatment. The rats were sacrificed on the 10^th^, 20^th^, 25^th^ and 45^th^ day of rotenone treatment. The caspase-3 mRNA and activated caspase-3 levels were evaluated and compared among one injection, two injections, three injections and four injections of NPs groups. Rotenone treatment would damage neurons in brain and therefore influence the body function. The changes of body weight could reflect the toxicity induced by rotenone treatment. The body weights decreased as the days of treating increased and were lower than initial body weights in rotenone treated group (**Fig. S4 in Supporting Information S1**). Meanwhile, the body weights were observed a continuous increase in oil treated group. The DPR/pshC-3 NPs injected group showed an increase in body weight compared with the other rotenone treated groups after the 34^th^ day.

Caspase-3 mRNA levels in brain were measured in rats injected with different NPs on specific days by real time PCR (RT-PCR) quantitatively (**Fig. 3A**). The mRNA level decreased and maintained below 50% compared to that in untreated control rat brains since the 20^th^ day after two injections of DPR/pshC-3 NPs. Meanwhile, the mRNA levels in DPR/pshSc treated and saline treated groups increased during the 10^th^ and 25^th^ day of rotenone treatment compared to that in untreated control rat brains. On the 45^th^ day of rotenone treatment, the level of caspase-3 mRNA of DPR/pshC-3 NPs injected group was lower than other groups. The results demonstrated that the mRNA level could decrease and maintain at a relatively low level after multiple dosing administration of caspase-3 shRNA encoding plasmid loaded brain-targeted peptide RVG29 modified NPs since the early stage of rotenone treatment. The scrambled shRNA loaded NPs failed to affect the level of caspase-3 mRNA which could prove that the caspase-3 shRNA sequence designed in this study was specific and effective. The non-targeted vector DGLs-PEG was also unable to deliver the caspase-3 shRNA encoding plasmid into the brain and interfere in the caspas-3 mRNA levels obviously.

The activated caspase-3 level was measured by western blot (**Fig. 3B**). The activated caspase-3 increased as the days of rotenone treatment increased since the 10^th^ day of rotenone treatment in saline and DPR/pshSc NPs injected rat brains. However, the activated caspase-3 remained at a low level by reduplicative DPR/pshC-3 NPs administration. The non-targeted NPs (DP/pshC-3) were again proved to have little effect on the activated caspase-3 level.

The activated caspase-3 was also compared by immunofluorescence assay in rats with rotenone treatment for 45 days (**Fig. 3C**, and **Fig. S5** and **Fig.** **S6 of Supporting Information S1**). The activated caspase-3 signals became less in DPR/pshC-3 NPs injected group compared to other control groups. The above results indicated that DPR/pshC-3 NPs could mediate caspase-3 mRNA silencing in rotenone treated rat brain and down-regulate the amounts of activated caspase-3 in brain.

### Behavioral Analysis

**Figure 4:**
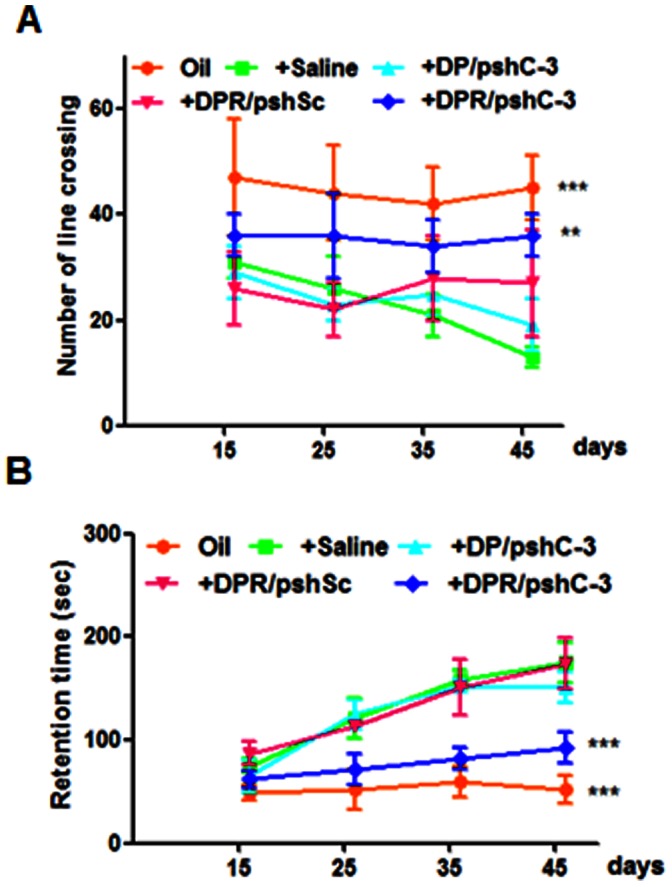
Behavioral changes of rotenone treated rats with different NPs injection. (**A**) Number of line crossing. (**B**) Retention time. Data are expressed as mean±S.E.M (n = 10). Significance: **p<0.01; ***p<0.001, significantly different as compared to saline injected rats with rotenone treatment (the negative control group).

During a multiple dosing regimen by weekly intravenous administration of different NPs since the 7th day of rotenone treatment, the time of inactive sitting (retention time) in DPR/pshC-3 NPs injected group remained at a low level and was similar to oil treated control group (**Fig. 4A**). Meanwhile, the retention time was longer in the other three groups including saline injected, DP/pshC-3 NPs injected and DPR/pshSc NPs injected groups. The number of line crossing in DPR/pshC-3 NPs injected group has been higher than other rotenone treated groups since the 15th of rotenone treatment (**Fig. 4B**).

### TH Immunohistochemical Staining

**Figure 5:**
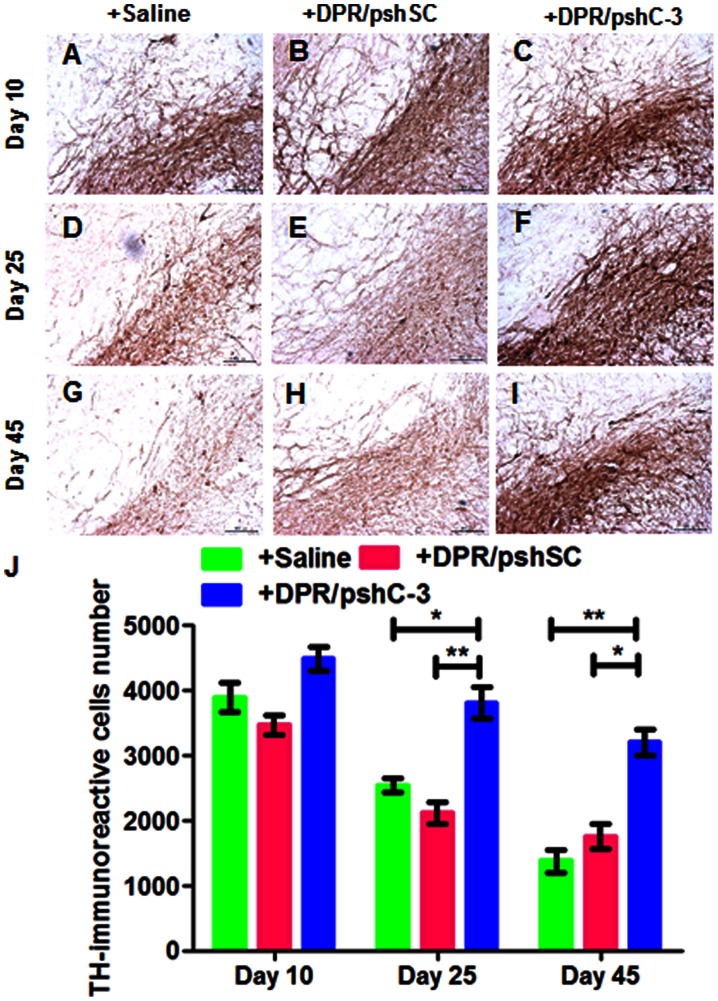
TH-immunoreactivity in the substantia nigra. Representative immuno-staining sections selected from saline (**A, D, G**) and different NP injected groups (DPR/pshSc NPs: **B, E H**; DPR/pshC-3 NPs: **C, F, I**) with rotenone treatment for (**A, B, C**) 10 days, (**D, E, F**) 25 days and (**G, H, I**) 45 days. (**J**) Stereological counting of TH positive nigral dopamine neurons following saline and different NP injected groups (DPR/pshSc NPs and DPR/pshC-3 NPs) with rotenone treatment for 10 days, 25 days and 45 days. * p<0.05; ** p<0.01.

As shown in **Fig. 5**, TH immunohistochemistry revealed that severe loss of dopaminergic cells in substantia nigra and fibers in striatum was found in rotenone treated non-injected group as well as DPR/pshSc NPs injected group. The sections of DPR/pshC-3 NPs injected group showed the most intensive TH-positive staining within the whole duration of rotenone treatment (**Fig. 5C, F, I**). The TH-immunoreactive neuron cells were counted using an optical fractionator method as shown in **Fig. 5J**. The result provided quantitative evidence demonstrating that repeated DPR/pshC-3 NPs injection could help to rescue TH-positive neurons in substantia nigra despite of rotenone treatment.

### Apoptosis Analysis by TUNEL

**Figure 6:**
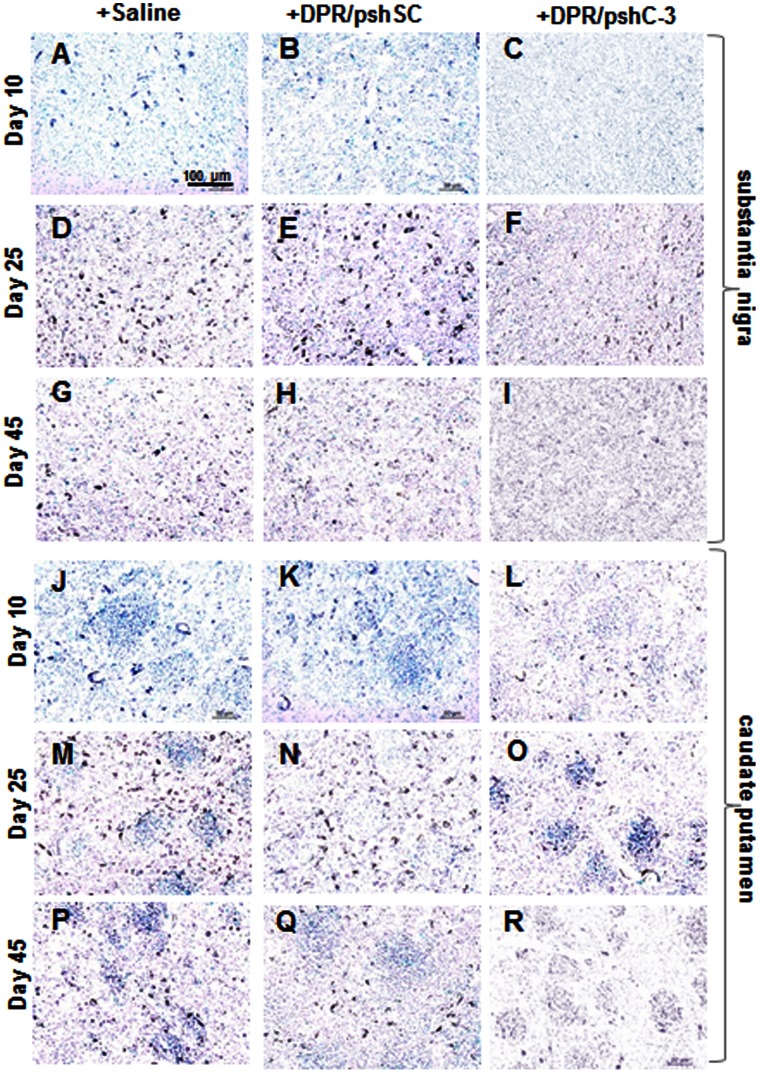
TUNEL staining on frozen sections of brain. (**A–I**) substantia nigra; (**J–R**) caudate putamen. Representative sections selected from saline and different NP injected groups with rotenone treatment for 10 days, 25 days and 45 days.

The neuron and microglia were two main components of brain parenchyma. The apoptosis was examined by TUNEL staining in brain sections on the 10^th^, 20^th^, 25^th^ and 45^th^ day of rotenone treatment with different NPs administration. In substantia nigra (**Fig. 6A–I**), the untreated or DPR/pshSc injected brain sections showed gradual increase in TUNEL-positive cells and more intensive TUNEL staining when compared with DPR/pshC-3 injected group from 10^th^ day to 25^th^ day. The positive staining in all the three groups (untreated, DPR/pshSc and DPR/pshC-3 injected) on the 45^th^ day decreased than that on 25^th^ day of rotenone treatment, respectively. The tendency of TUNEL-positive staining in striatum was similar to that in substantia nigra (**Fig. 6J–R**).

### TNF-α and NO Assay

**Figure 7:**
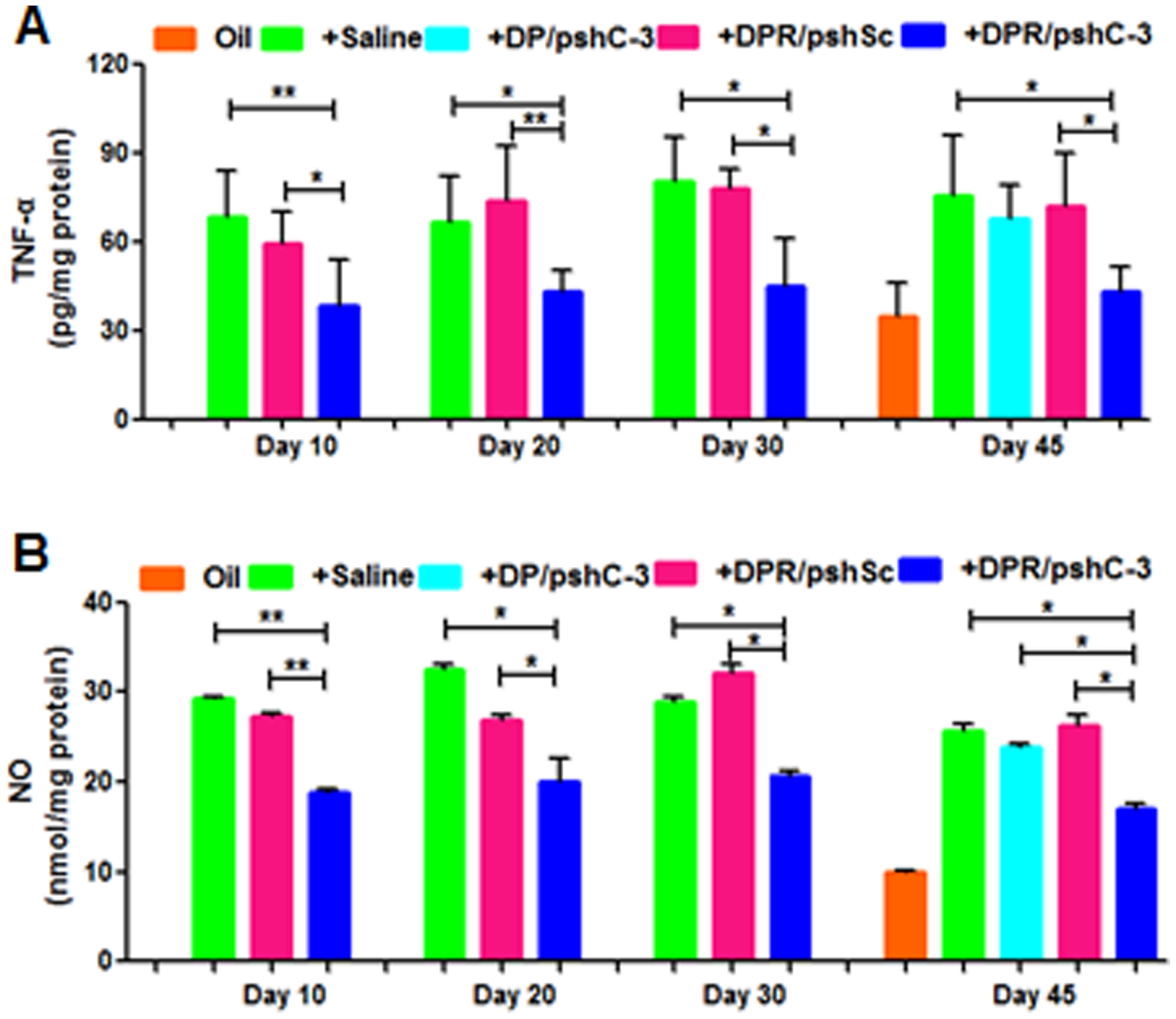
The effects of rotenone treatment on inflammatory related markers. (**A**) pro-inflammatory cytokine TNF-α level and (**B**) NO level were examined in midbrain tissues (mainly substantia nigra) with various length of rotenone treatment injected with saline or different NPs. Data are expressed as mean±S.E.M (n = 3). Significance: *p<0.05; **p<0.01, significantly different as compared between two groups.

The effects of rotenone treatment on pro-inflammatory cytokine (measure by TNF-α) and NO level were examined in midbrain tissues (mainly substantia nigra) with various length of rotenone treatment injected with different NPs. **Fig. 7A** showed continuous treatment of rotenone led to a significant and permanent increase in TNF-α level in brain tissues since the 10^th^ day of rotenone treatment. The TNF-α level reached its peak on about the 30^th^ day of rotenone treatment. Meanwhile, rotenone treatment failed to significantly affect the level of pro-inflammatory molecule TNF-α in DPR/pshC-3 NPs injected group since the 10^th^ day of rotenone treatment.

The NO level which reflected iNOS activity was greatly increased after 10 days of rotenone treatment in both non-injected and DPR/pshSc NPs injected groups (**Fig. 7B**). The NO level reached its maximum in midbrain between 20 days and 25 days after treatment of rotenone while it was decreased on the 45^th^ day of rotenone treatment. In DPR/pshC-3 NPs injected group, the level of NO remained lower than that of non-injected and DPR/pshSc NPs injected groups during the whole period of rotenone treatment.

## Discussion

Caspases could regulate apoptotic neuronal death as well as inflammation of the central nervous system [11], [24]. Till now, there is no available non-viral gene therapy approach targeted to caspases overcoming the presence of the blood-brain barrier (BBB). In this study, brain-targeted gene delivery nano-platform was applied for the treatment of rotenone-induced PD based on the hypothesis of anti-apoptotic and anti-inflammation as synergistic therapeutic benefits by down-regulating the expression and activation level of caspase-3 in brain. Herein, we chose the rotenone-induced PD rats as the in vivo model. This in vivo model of PD was believed to be more accurate to natural occurrence as it was chronic, progressive and had selective nigrostriatal dopaminergic degeneration and Lewy body formation [6]. Furthermore, rotenone exposure reproduced the glial pathology observed in PD: selective and extensive microglial activation in the nigrostriatal pathway [25]. The above two main features made the rotenone-induced PD model an ideal model for our hypothesis that explored the possibility of anti-apoptotic and anti-inflammation as synergistic therapeutic benefits by down-regulation the expression and activation level of caspase-3. In our previous study, we demonstrated rotenone treatment wouldn’t affect the BBB integrity in in vitro BBB model. In addition, the BBB functionality didn’t change in Parkinson’s disease model induced by rotenone [26]. The RVG29 modified nanoparticles possessed a rapid and efficient accumulation in brain since 15 min after administration compared to unmodified and non-treated groups (**Fig. 1** **and** **Fig. 2G**). Once recognizing and binding to its specific receptor in the brain capillary endothelial cells, the brain-targeted nanoparticles would get across the BBB and enter the brain parenchyma (mainly composed by neurons and microglia) (**Fig. 2**). Previous studies also proved the RVG29 modified drug delivery systems could get into the brain through specific ligand-receptor mediated transcytosis [17]. The nanoparticles could be further uptake by neural cells or microglia. Thus, the level of activated caspase-3 could decrease in neural cells and microglia in brain-targeted nanoparticles injected rat brain.

The low level of activated caspase-3 could protect the neurons in the presence of rotenone. Neurons expressing caspase-3 were more sensitive to the pathological process than those that do not express the protein [9]. The anti-apoptosis effect was confirmed by TUNEL staining. The distribution of TUNEL staining was consistent with the previous research that the obvious symptoms of PD did not develop until there was an estimated 50–60% loss of DA neurons in the substantia nigra [27]. It also certified that early prevention and therapy was crucial to PD treatment.

The anti-inflammation effect of low activated caspase-3 level in microglia was confirmed by the measuring the pro-inflammatory cytokine (TNF-α) and NO levels in rats midbrain tissues (mainly substantia nigra) with various length of rotenone treatment and injected with different NPs. Chronic treatment of rotenone over a long period was capable of increasing NO and mimic Parkinson’s disease (PD)-like behavioral symptoms that were akinesia and rigidity in rats [28]. The results indicated that repeated administration of brain-targeted NPs loading anti-Caspase-3 shRNA encoding plasmid could decrease the TNF-α and NO level induced by rotenone treatment.

### Conclusions

The hypothesis of anti-apoptotic and anti-inflammation as synergistic therapeutic benefits by down-regulating the expression and activation level of caspase-3 in brain was demonstrated. Obvious neuron rescue was found in group injected with DPR/pshC-3 NPs. Meanwhile, the behavioral results suggested the locomotor activity of rats could be improved by prompt and efficient caspase-3 down-regulation and prevention of neuron death at the early stage of the disease. So far, there have been few effective drugs targeting to inhibit or down-regulate caspase-3 in neurodegenerative diseases in clinic. The non-viral caspase-3 RNAi delivery system targeting to the brain would hold great promise for further application. Meanwhile, it is also necessary to evaluate the long-term toxicity and side effects.

## Supporting Information

Supporting Information S1 This supplemental file contains the following: This supplemental file contains the following: Supporting Materials and Methods. Figure S1: Characterization of the NPs. Figure S2: Caspase-3 mRNA silencing percentage by RT-PCR in SH-SY5Y cells using different caspase-3 shRNA encoding plasmid. Figure S3: In vitro toxicity evaluation by MTT. Figure S4: The body weight changes during rotenone/oil treatment with weekly administration of different NPs. Figures S5 and S6: Immunofluorescence images of activated caspase-3 during the treatment of rotenone for various days with different NPs in different rat brain regions.
(DOC)
