# Correction: Targeting Caspase-3 as Dual Therapeutic Benefits by RNAi Facilitating Brain-Targeted Nanoparticles in a Rat Model of Parkinson’s Disease

**DOI:** 10.1371/annotation/5f08fe1e-8868-421c-92ea-1a4aa987d11f

**Published:** 2013-09-04

**Authors:** Yang Liu, Yubo Guo, Sai An, Yuyang Kuang, Xi He, Haojun Ma, Jianfeng Li, Jing Lu, Ning Zhang, Chen Jiang

There was an error in the name of the eighth author.

The correct name of this author is: Jing Lu 

