# Supplementary material for: Targeting Caspase-3 as Dual Therapeutic Benefits by RNAi Facilitating Brain-Targeted Nanoparticles in a Rat Model of Parkinson’s Disease
Source: PLoS One. 2013 May 13;8(5):e62905. doi: 10.1371/journal.pone.0062905 (PMC3652845; doi:10.1371/journal.pone.0062905)
Supplement: Supporting Information S1 — This supplemental file contains the following: This supplemental file contains the following: Supporting Materials and Methods. Figure S1: Characterization of the NPs. Figure S2: Caspase-3 mRNA silencing percentage by RT-PCR in SH-SY5Y cells using different caspase-3 shRNA encoding plasmid. Figure S3: In vitro toxicity evaluation by MTT. Figure S4: The body weight changes during rotenone/oil treatment with weekly administration of different NPs. Figures S5 and S6: Immunofluorescence images of activated caspase-3 during the treatment of rotenone for various days with different NPs in different rat brain regions. (DOC) [file pone.0062905.s001.doc]

**Supporting Information**

1. **Preparation and characterization of NPs**

DGLs (containing 123 primary amino groups, generation 3; COLCOM, France) was reacted with NHS–PEG3400–MAL (JenKem Technology, China) at the ratio of 1:10 (mol/mol) in PBS (pH 8.0) for 2 h at room temperature. The primary amino groups on the surface of DGLs were specifically reacted with the NHS groups of the bifunctional PEG derivative. The resulting conjugate, DGLs-PEG, was purified by ultrafiltration through a membrane (cutoff 5 kDa) and the buffer was changed to PBS (pH 7.0). Then DGLs-PEG was reacted with peptide RVG29, 1:1 (mol/mol) in PBS (pH 7.0) for 24 h at room temperature. The MAL groups of DGLs-PEG were specifically reacted with the thiol groups of RVG29. The successful synthesis of DGLs-PEG-RVG29 was confirmed by NMR spectra (shown in **Fig. S1A**).

Dendrimers (DGLs-PEG or DGLs-PEG-RVG29) were freshly prepared and diluted to appropriate concentrations in PBS (pH 7.4). DNA solution (100 mg DNA/ml 50 mM sodium sulfate solution) was added to obtain specified weight ratios (6:1, DGLs to DNA, w/w) and immediately vortexed for 30 s at room temperature. Freshly prepared NPs were used in the experiments that follow. The mean diameter of DPR/DNA NPs was determined by dynamic light scattering (DLS) and transmission electron microscope (TEM). The results (shown in **Fig. S1B and C**) indicated that the DPR/DNA NPs were spherical particles with a hydrated diameter of 97nm. The ability of plasmid encapsulation as well as the stability of NPs loading DNA against enzymes digestion was confirm by agarose gel electrophoresis. No DNA release was observed at the weight ratio of DGLs to DNA used in this experiment. The optimization of the weight ratio was discussed in our previous study.


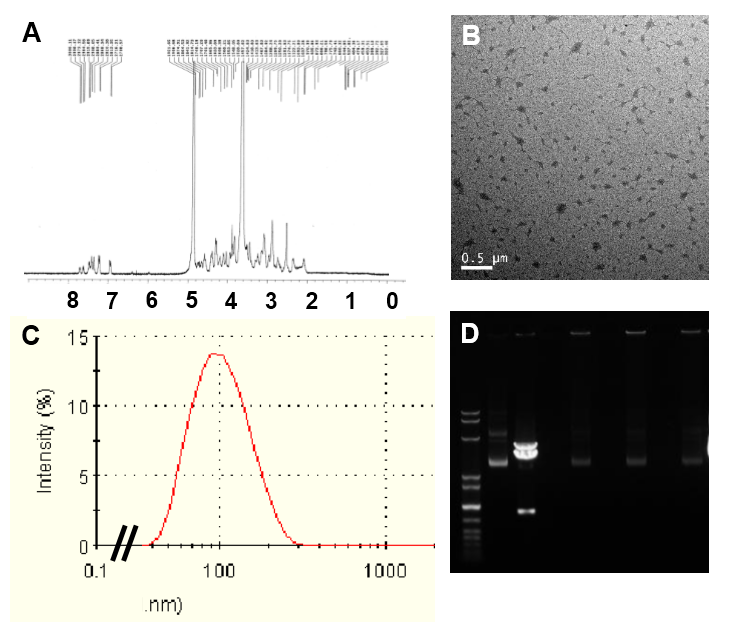


**Fig. S1.** Characterazation of DPR and DPR/DNA NPs. (**A**) NMR spectra of DGLs-PEG-RVG29 in D_2_O at 400 MHz. (**B**) TEM image of DPR/DNA NPs. (**C**) The particle size distribution of DPR/DNA NPs determined by DLS. (**D**) Agarose gel electrophoresis evaluation of DNA encapsulation and protection of NPs. Lane 1: marker; lane 2: naked DNA; lane 3: naked plasmid DNA treated with enzymes; lane 4: DGLs/DNA NPs; lane 6: DP/DNA NPs and lane 8: DPR/DNA NPs. The stability of NPs loading DNA against enzymes digestion. Plasmid DNA was released from the NPs by the addition of sodium heparin separated by agarose gel electrophoresis after enzymes incubation. lane 5,7 and 9: DGLs/DNA, DP/DNA and DPR/DNA NPs with treatment of heparin after enzymes incubation.

1. **Caspase-3 shRNA encoding plasmid selection**

Three different caspase-3 shRNA sequence were designed according to the results by applying specific software shown in the following table.

| **Name** | **Sense strand 5’ to 3’** | **T_m_(℃)** |
| --- | --- | --- |
| pSc | caccGTTCTCCGAACGTGTCACGTcaagagattacgtgACACGTTCGGAGAAttttttg | 121 |
| pshC-3-1 | caccGCAGTTACAAAATGGATTATtcaagagATAATCCATTTTGTAACTGCttttttg | 104 |
| pshC-3-2 | caccGCCGACTTCCTGTATGCTTACTtcgaagagAGTAAGCATACAGGAAGTCGGCttttttg | 114 |
| pshC-3-3 | caccGCCGAAACTCTTCATCATTCATtcaagagATGAATGATGAAGAGTTTCGGCttttttg | 116 |

All the four shRNA encoding plasmids were encapsulated with DPR yielding different NPs. The SH-SY5Y cells were incubated with different NPs for 48h in 6 well cell culture plate. The gene silencing efficiency was evaluated by RT-RCR (methods would be mentioned in other part in this manuscript). Among all the three caspase-3 shRNA encoding plasmid, the pshC-3-3 sequence showed the most gene silencing effect (seen the following figure). This sequence was selected for the further experiments. Meanwhile, the scramble sequence showed little effect on changing the caspase-3 mRNA level. It would be used as negative control.


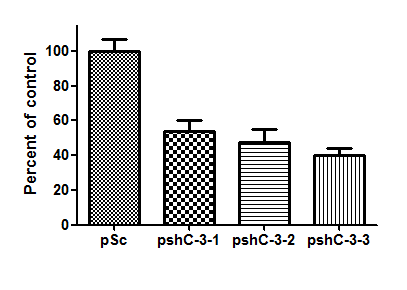


Fig. S2. Caspase-3 mRNA silencing percentage by RT-PCR in SH-SY5Y cells using different caspase-3 shRNA encoding plamid. Data are expressed as mean±S.E.M (n=3).

1. **Cell culture and *in vitro* toxicity evaluation**

SH-SY5Y human neural cells (ACTT No. CRL-2266) were gifts from Prof. L.Y. Feng (Shanghai Institute of material medica, Chinese academy of sciences). SH-SY5Y were cultured in DMEM, supplemented with 10% fetal bovine serum (FBS), 100 U/ml penicillin and 100 mg/ml streptomycin at 37 °C in a humidified 5% CO2 incubator. Brain capillary endothelial cells (BCECs) were kindly provided by Prof. J. N. Lou (the Clinical Medicine Research Institute of the Chinese-Japanese Friendship Hospital). Briefly, BCECs were expanded and maintained in special Dulbecco’s modified Eagle medium (Sigma-Aldrich) supplemented with 20% heat-inactivated fetal bovine serum (FBS), 100 μg/ml epidermal cell growth factor, 2 mmol/L l-glutamine, 20 μg/ml heparin, 40 μU/ml insulin, 100 U/ml penicillin, and 100 μg/ml streptomycin. All cells used in this study were between passage 9 and passage 20.

The cytotoxicity of the DLGs, DP/DNA and DPR/DNA NPs at different concentrations was evaluated in BCECs and SH-SY5Y cells by MTT assay. As shown in Fig. S2, the cell viability of the three NPs at the concentrations within 200μg/ml (calculated by DGLs) was above 80% in both BCECs and SH-SY5Y cells which was thought to be safe enough in cells. Meanwhile the cell viability showed no significant difference at both 2 h and 48 h. The circulating blood volume of rats was estimated to be 15ml. The dose of DGLs injected in each rat was 600μg. Thus, the concentration of NPs in circulation was about 40μg/ml (calculated by DGLs) which was much lower than 200μg/ml. The results demonstrated the DGLs showed high biocompatibility.


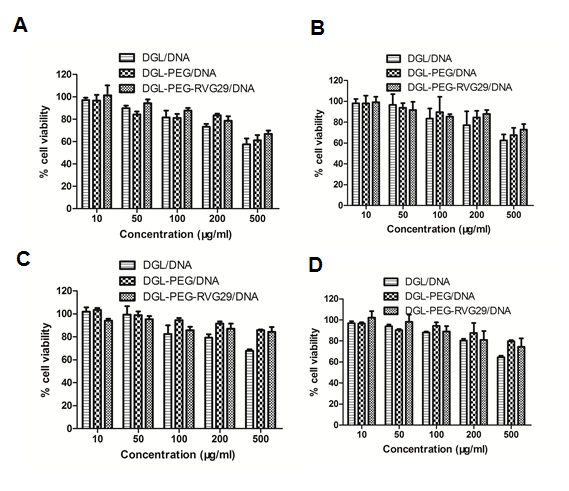


**Fig. S2.** *In vitro* toxicity evaluation by MTT. (**A**) The BCECs viability with the incubation of DGLs/DNA, DP/DNA and DPR/DNA NPs at different concentrations with 2 h incubation. (**B**) The BCECs viability with the incubation of DGLs/DNA, DP/DNA and DPR/DNA NPs at different concentrations at 48 h after the 2 h incubation and removal of NPs. (**C**) The SH-SY5Y cells viability with the incubation of DGLs/DNA, DGLs-PEG/DNA and DPR/DNA NPs at different concentrations with 2 h incubation. (**D**) The SH-SY5Y cells viability with the incubation of DGLs/DNA, DGLs-PEG/DNA and DPR/DNA NPs at different concentrations at 48 h after the 2 h incubation and removal of NPs.

1. **The changes of body weight during rotenone treatment and NPs administration**


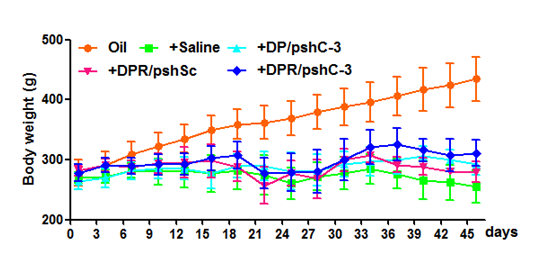


**Fig. S3.** The body weight changes during rotenone/oil treatment with weekly administration of different NPs.

1. **The activated caspase-3 immunofluorescence assay in rats with rotenone treatment for 45 days**


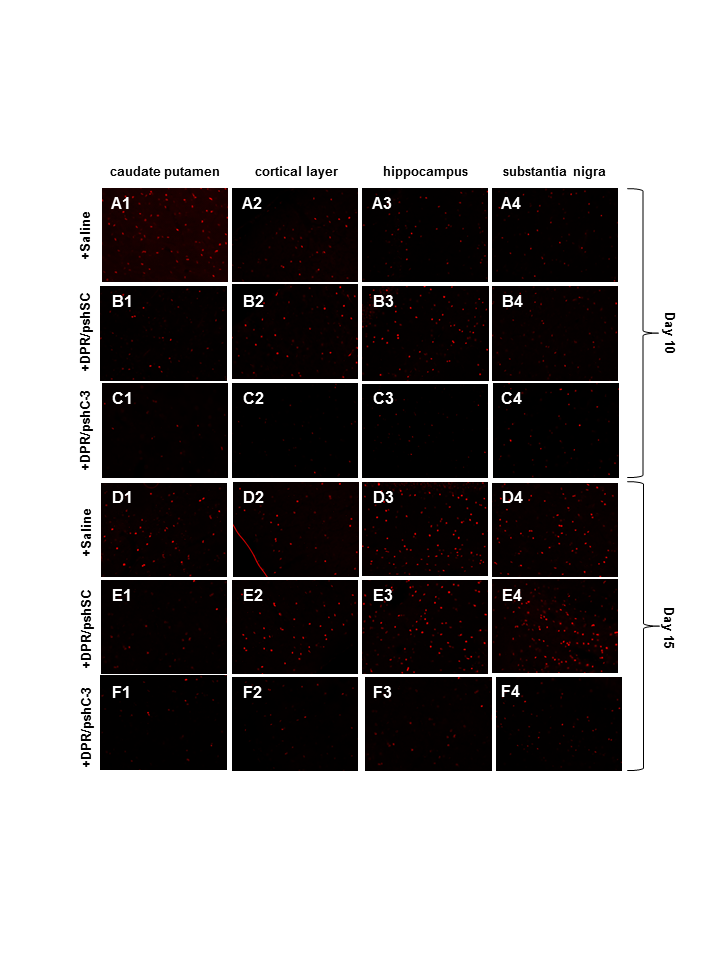

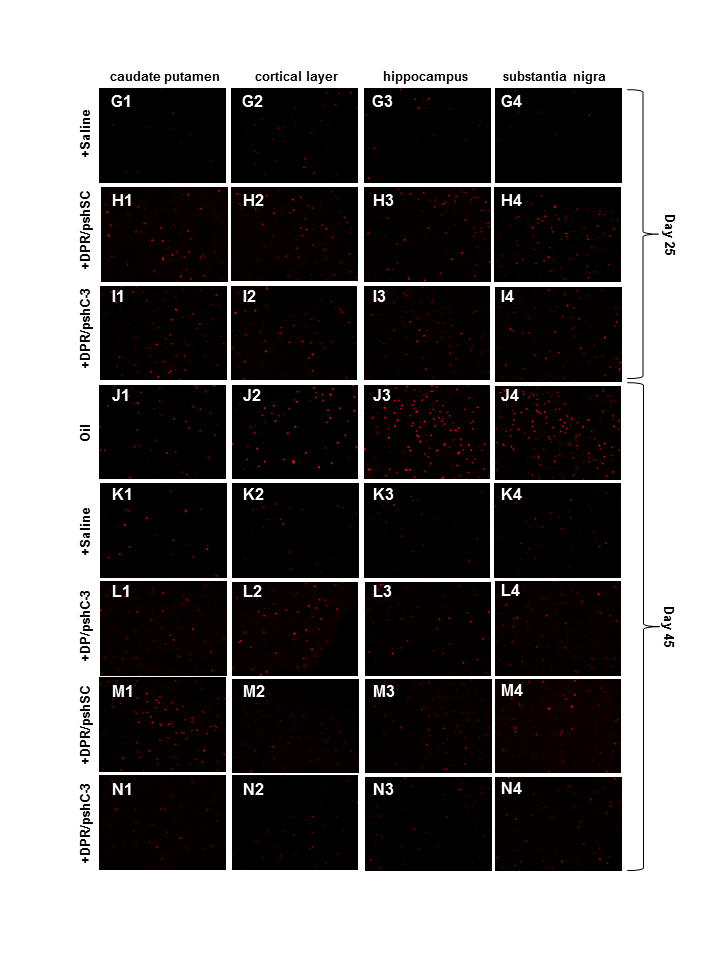


**Fig. S4** Immunofluorescence images of activated caspase-3 during the treatment of rotenone for various days with different NPs in different rat brain regions. Red: Alexa Flour 555 secondary antibody labeled activated caspase-3. Original magnification: ×200.


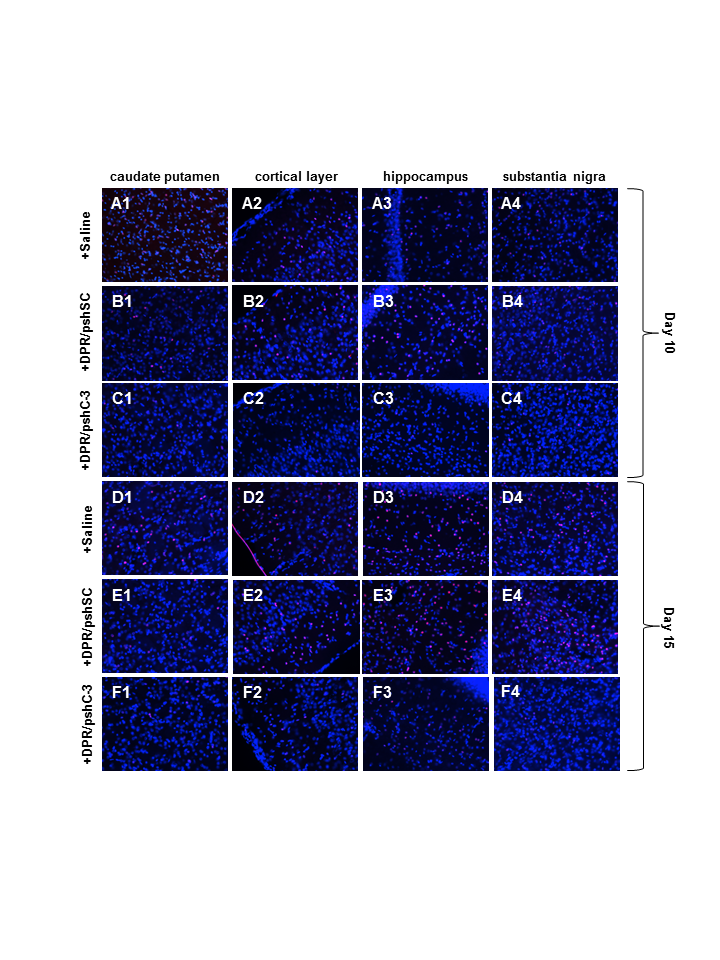


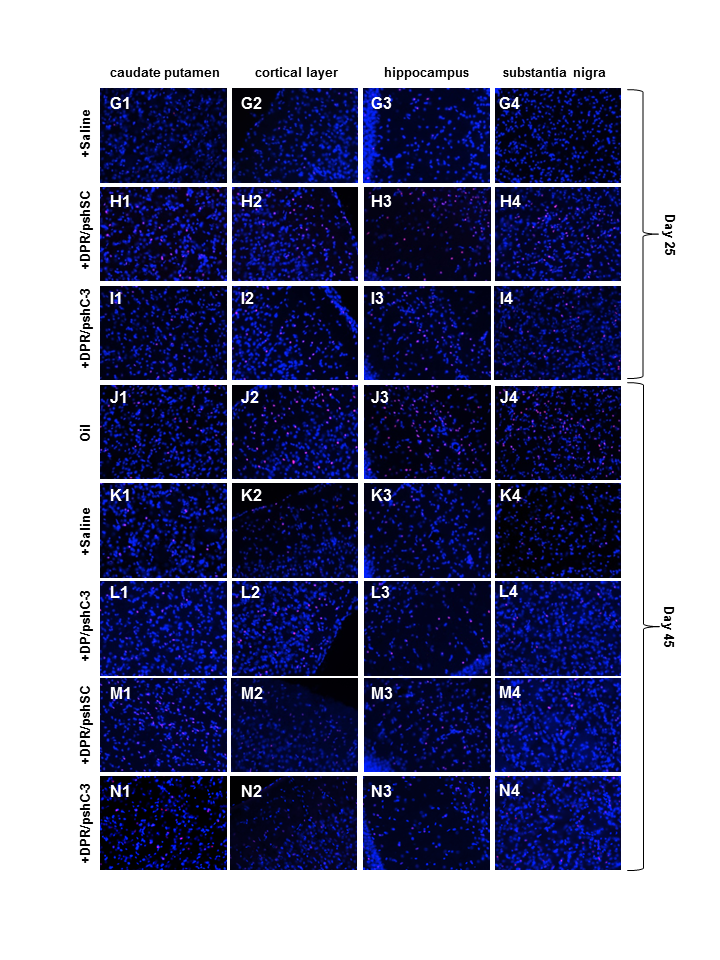


**Fig. S5.** Overlay images of activated caspase-3 immunofluorescence and nuclei during the treatment of rotenone for various days with different NPs in different rat brain regions. Red: Alexa Flour 555 secondary antibody labeled activated caspase-3. Blue: DAPI stained nuclei. Original magnification: ×200.
